# Long-term detoxification of opioid use disorder with opium tincture assisted treatment

**DOI:** 10.3389/fpsyt.2023.1273587

**Published:** 2023-12-07

**Authors:** Sahar Omidvar Tehrani, Amir Rezaei Ardani, Saeed Akhlaghi, Masood Shayesteh Zarrin, Ali Talaei

**Affiliations:** ^1^Psychiatry and Behavioral Sciences Research Center, Mashhad University of Medical Sciences, Mashhad, Iran; ^2^Department of Psychiatry, Southwest Centre for Forensic Mental Health Care, Schulich School of Medicine & Dentistry, University of Western Ontario, London, ON, Canada; ^3^Department of Biostatistics, School of Health, Mashhad University of Medical Sciences, Mashhad, Iran

**Keywords:** relapse, craving, opioid use disorder, detoxification, opium tincture

## Abstract

**Introduction:**

Retention in treatment, craving, and relapse rate are important indicators of the success rate in addiction maintenance therapy as they evaluate the effectiveness of the therapy and make necessary adjustments to the treatment plan. However, the rate of continuation in the treatment process and the rate of craving in patients with opioid use disorder undergoing maintenance treatment with opium tincture have not been studied. The present study aimed to investigate the rate of relapse, craving, and psychiatric disorders in patients with opioid use disorder undergoing treatment of gradual detoxification with opium tincture.

**Methods:**

Ninety patients with opioid use disorder who underwent treatment with the gradual detoxification method using opium tincture in the form of Congress 60 for 11 months were enrolled in the study. The level of craving based on the Desire for Drug Questionnaire (DDQ) and patients’ self-report of drug use, the level of anxiety, depression, and sleep quality of patients were evaluated using the Beck Anxiety Inventory (BAI), Beck Depression Inventory (BDI), and Pittsburgh Sleep Quality Index (PSQI), respectively. Also, suicidal thoughts were assessed by Beck Scale for Suicidal Ideation and quality of life by the World Health Organization Quality of Life Questionnaire (WHOQOL-BREF).

**Results:**

The study found that the treatment resulted in a relapse rate of 16.7% for relapse. We also found that all sub-scales of the desire for drug questionnaire (DDQ), depression, and anxiety were significantly lower at the end of the study compared to its beginning. Additionally, quality of life and sleep significantly increased at the end of the study. All areas of craving, anxiety, and depression significantly decreased in all follow-up sessions that took place 1, 5, and 11 months after the start of treatment. Moreover, sleep disorders were improved considerably at the end of the treatment.

**Conclusion:**

The current study presented a low relapse rate of Iranian patients with opioid use disorder under structured treatment of gradual detoxification with opium tincture in a one-year follow-up period. Opium tincture under the Congress 60 protocol may help to control carving, decrease psychological disorders, improve quality of life, and consequently, lower relapse rate.

## Introduction

Substance use disorders are a significant public health concern and a leading cause of the global burden of disease (GBD) in both developed and developing countries (1). Substance use is a major contributor to the disease burden in the Iranian population (2), with a higher prevalence of opioid use among individuals over 15 years old, 5.4% (3), compared to the global average of 0.8% (4). According to the latest census, the number of individuals dependent on drugs in Iran ranges from 1.2 and 2 million, of which 9–16% are injecting drug users (5). Due to the high prevalence of opioid use disorder in Iran, various treatment options, such as maintenance treatment, have been proposed. Maintenance treatment involves the substitution of illegal drugs, such as heroin and opium, with opioid agonists like methadone and buprenorphine. This approach has become one of the primary treatment methods in recent decades. It should be noted that in Iran methadone and buprenorphine must be prescribed in licensed outpatient treatment programs only that does not include other forms of therapy or support, this limits access to opioid agonist treatment protocols and packages. Therefore, Congress 60 has been highlighted as an alternative maintenance therapy that includes psychosocial therapy, education and group therapy as well.

Opium tincture was developed as a harm reduction strategy by the Ministry of Health to control opioid use disorder, the most consumed drug in Iran (6). This compound has been accepted as an alternative to drugs, such as methadone, for detoxification and maintenance treatment of opioid use disorder and relief of drug withdrawal symptoms in certain parts of the world (7). The tincture of opium is a clear, reddish-brown hydroalcoholic solution of opium with a characteristic odor and bitter taste. It is offered in two forms, 1 and 2%, and it is an alcoholic extract (20%) of opium. The active substance of opium tincture is Morphine (C17H19NO3), which is standardized to contain 1% morphine. Since its introduction in 2010, opium tincture has been used on over 94,000 patients (8), and it is the second most common drug after methadone for the maintenance treatment of opioid use disorder (9).

Long-term treatment with opium tincture can be administered in two ways, one as a maintenance treatment or self-administered gradual dose reduction, and another is a combination of self-help group programs in non-governmental organizations that include peer counseling and recreational activities, the latter of which has gained significant popularity in recent years (10). One NGO is Congress 60, established in 1998 and has grown to 38 branches in Iran with more than 20,000 members. The treatment in Congress typically lasts 10 to 11 months and involves gradual dose reduction over 21 days intervals until the substance use is eliminated. Congress 60 program consists of the DST method (an 11-month process of drug tapering using opium tincture) combined with intensive psychosocial support provided within a philosophy of recovery. Congress 60 offers educational classes and group therapy to promote positive thinking and mental balance (11, 12).

Zarghami et al. stated that because of historical, cultural and geographical use and production of opium in the middle east region, use of opium tincture as a treatment for opium use disorder is less stigmatized relative to alternative treatments and results in more compliance to treatment among patients (13). Moreover, it is shown that due to cultural misconception about methadone, opium addicts are more inclined to use opium tincture as a treatment (14). In regard to psychotherapy and support group session, this aspect of Congress 60 is similar to Alcoholic Anonymous (AA) and run by volunteers. However, it should be noted that opium tincture itself and its administration is not hundred percent subsidized, though relatively a lot more cost effective option compared to other alternatives.

In maintenance treatment programs, the success rate is measured by reducing the physical, family, and social harms. However, since substance use disorder is a chronic and relapsing disorder, some patients continue to use illegal drugs during treatment. The retention rate in maintenance therapy is one of the important indicators of treatment success. However, the rate of continuation in the treatment process and the rate of craving in patients undergoing maintenance treatment with opium tincture (as a pure opioid compound) have not been studied. To address this gap, the present study was designed to investigate the relapse rate, craving, and psychiatric disorders in opioid users undergoing structured treatment of gradual detoxification with opium tincture.

## Methods

### Ethics statement

This research was approved by the ethics committee of Mashhad University of Medical Sciences with the ethical code IR.MUMS.MEDICAL.REC.1399.686, all the study steps were carried out according to ethical protocols.

Informed consent was obtained from all participants before entering the study and completing the questionnaires, and after the start, they could voluntarily withdraw from the study. Also, in completing the questionnaires, the identity information of the participants was not recorded, and other information was obtained confidentially and only for the research, without mentioning names. Each questionnaire had a code. The code related to the patient was recorded in a separate book so that if there were a need to give feedback to the patients based on any of the questionnaires, it would be possible to access their names. If the answer to the questionnaire were one of the life-threatening cases, such as severe depression and suicidal thoughts, they would be informed and referred for treatment.

### Study population

This study was conducted using a purpose-based sampling method. Totally, 90 individuals with opioid use disorder were verified by the DSM-5 diagnostic criteria and were enrolled in the study. The patients were treated with the gradual detoxification method with opium tincture in the form of Congress 60 at a treatment center for substance use disorders and addictive behaviors in Mashhad and the addiction treatment clinic of Ibn-e-Sina Psychiatry Hospital, Mashhad University of Medical Sciences, Mashhad, Iran, between March 2021 and July 2022.

### Inclusion and exclusion criteria

Patients were diagnosed based on DSM-5 diagnostic criteria; they were aged between 18 and 64 years, with only opioid use disorder, and without a history of major psychiatric disorders (including bipolar disorder, schizophrenia, and schizoaffective disorder).

Exclusion criteria for this study were: unwillingness to continue participating in the study, unwillingness to continue treatment with opium tincture in the form of Congress, and contraindications to the use of opium tincture due to medical illness.

### Study procedure

At first, patients with opioid use disorder who chose the gradual detoxification method with opium tincture in the form of Congress 60 were assured that their identity information would not be recorded and would remain confidential. It was also briefly explained in simple language about this study’s importance, future applications, and the time required to complete the questionnaires. In the case of informed acceptance, the psychiatry resident evaluated the patient for opioid use disorder based on DSM-5 using a diagnostic interview. If there are inclusion and exclusion criteria, demographic and clinical information was collected by clinical interview. During the follow-up some participants (12 patients) chose to opt out of the study – though continuing the treatment. At the end of the study 78 of participants remained in the study.

The study evaluated several factors, including the level of craving and drug use tendency (measured by DDQ), relapse assessed by patient’s self-report of drug use and psychiatry interview conducted by psychiatry resident, anxiety, depression, and sleep quality of patients assessed by Beck anxiety, Beck depression, and Pittsburgh sleep quality questionnaires, respectively. Also, suicidal thoughts were assessed by Beck’s suicide scale questionnaire and quality of life by the WHO quality of life questionnaire.

Under the treatment program of Congress 60, individuals are treated with a gradual reduction of opium tincture for 11 months. Following the guideline of Congress 60, with the permission of the Ministry of Health, within the first month, the substance consumed by the patient will be converted into opium tincture. Within this first month, opium tincture can be consumed alongside the patient’s substance of use. In this first month, the opium tincture reaches a maximum dose of 16.5 mL, and then, the drug dose is reduced by 1.65 mL every 3 weeks (21 days) so that after 11 months, the drug dose reaches zero. Congress 60 protocol also includes psychosocial support, group therapy and educational classes. All the captured data is under the Congress 60 protocol.

The patients were assessed by a psychiatry resident and psychologist at five different time points: the time of arrival, 1 month after the start of treatment, 5 months after the start of treatment, immediately after the end of treatment, and 1 month after the end of treatment.

### Instruments

#### Demographic and clinical checklist

Items, including age, gender, marital status, employment status, education, history of suicide, history of suicide in the family, history of relapse, age of starting opioid use, history of smoking, history of psychiatric disorder, history of psychiatric illness in the family, medical history was obtained using interview and filling out the demographic and clinical checklist.

#### The desire for drug questionnaire

DDQ is a 13-question questionnaire designed in 2002 by Franken et al. This questionnaire is derived from the alcohol craving questionnaire used for heroin addicts. However, the ability to measure the overall substance has also been used to measure cravings for other substances. This questionnaire has three subscales, desire and intention to use drugs (8 questions), negative reinforcement (4 questions), and control over drug use (2 questions). The scoring of the questionnaire is based on a 7-point Likert scale (completely disagree to completely agree). Completely disagree receives a score of one, and completely agrees gets a score of seven. Franken and colleagues have reported the overall validity of this questionnaire as 0.85 Cronbach’s alpha and 77, 80, and 75% for its subscales, respectively (15). In the research of Hassani et al., the total Cronbach’s alpha value was reported as 0.82 and its subscales as 0.89, 0.79, and 0.4, respectively (16).

#### Beck Depression Inventory

Aaron Beck and colleagues designed this questionnaire in 1961 (17). BDI is a 21-item multiple-choice self-report questionnaire used to measure the severity of depression in adolescents and adults in the general population and people with depression. Each question gets a score between 0 (no mark) and 3 (severe). These questions include mood, physical, cognitive, and vegetative symptoms but do not include anxiety. Then the scores are summed up and presented from zero to 63. Scores of 0–21 are expressed as mild, 22–31 as moderate, and 32–63 as severe (18). This questionnaire has a good internal correlation with Cronbach’s alpha of 0.93. The validity and reliability of this questionnaire in the Persian version are 0.73 and 0.91, respectively (19).

#### Beck Anxiety Inventory

It was designed by Aaron Back et al. in 1988. The Beck Anxiety Questionnaire is a 21-item multiple-choice self-report questionnaire used to measure anxiety intensity in adolescents and adults. Each question gets a score between 0 (no mark) and 3 (severe). Then the scores are summed and presented from 0 to 63. Scores of 0–21 are expressed as mild, 22–35 as moderate, and 36–63 as severe. The internal correlation with Cronbach’s alpha is 0.92 (20). The validity and reliability of this questionnaire in the Persian version are 0.72 and 0.83, respectively (21).

#### Pittsburg Sleep Quality Index

It was designed by Dr. Daniel J. Bice and colleagues in 1989. This questionnaire measures the quality of sleep over 1 month and includes 19 items, seven of which are sleep characteristics, including subjective sleep quality, delay in falling asleep, sleep duration, sleep disorders, sleep habits, and use of sleeping pills; and it measures dysfunction during the day. Based on severity, each of these seven items is given a score of 0 to 3, so the range of scores will be between 0 and 21. A higher score the patient receives indicates a lower sleep quality, so a score higher than five is considered a major sleep disorder. The reliability of this scale is 0.83, and the creators of this scale have reported its validity with 89.6% sensitivity and 86.5% specificity at an appropriate level (22). In Farrahi et al. study, internal correlation with Cronbach’s alpha of 0.89 and sensitivity and specificity were reported as 100 and 93%, respectively (23).

#### Beck Scale for Suicidal Ideation (BSSI)

It was designed by Beck et al. in 1988. The BSSI questionnaire is a 19-item self-report questionnaire that measures the presence and intensity of suicidal thoughts in the previous week. Each question gets a score between 0 and 2, and the total score ranges from 0 to 38. A score of 0–5 indicates low risk, 6–19 moderate to high risk, and 20–38 very high risk for suicide (24). The overall Cronbach’s alpha rate is 0.95, sensitivity is 75%, and specificity is 88.9% (25). In the research of Esfahani and other colleagues, the internal correlation with Cronbach’s alpha was reported as 82% (26).

#### The World Health Organization Quality of Life Questionnaire

It is a 26-item questionnaire that includes four areas of physical health (7 items), mental health (6 items), social relations (3 items), and environmental health (8 items). The first two questions do not belong to these areas and measure health status and quality of life in general. Each item has a score between 1 and 5. The internal correlation of this test with Cronbach’s alpha is more than 0.7. This rate was reported as 0.82, 0.81, 0.80, and 0.68 in physical health, mental health, environmental health, and social relations, respectively (27). In Nejat et al.’s study, Cronbach’s alpha was more than 0.7, and the validity and reliability of the test were reported as favorable (28).

### Statistical analysis

The data were analyzed using SPSS version 16, with a significance level of *p* < 0.05. Descriptive statistics were used to illustrate study population characteristics. An independent *t*-test and ANOVA were used to compare mean scores between two-time points and over time. Nonparametric tests were applied when the data did not follow a normal distribution.

## Results

The study population characteristics are summarized in Table 1. The average age of the patients was 37.74 with a standard deviation of 7.93 years, they constituted 14.4% females and 85.5% males, and 82.2% of them were married. Examining the drug use history showed that 95.6% had a history of opium use, and 83.3% had a history of shireh (opium juice) use.

**Table 1:** The characteristics of the study population.

Characteristics	Group	Number (%)
Age*		37.74 ± 7.93
Sex	Male	77 (85.5)
	Female	13 (14.4)
Marital status	Single	11 (12.2)
	Married	74 (82.2)
	Widow/deceased	5 (5.6)
Education	Illiterate	4 (4.4)
	High school	41 (45.6)
	Diploma/associate	39 (43.3)
	Bachelor	4 (4.4)
	Master/doctorate	2 (2.2)
Employment status	Unemployed	21 (23.3)
	Full time employee	49 (54.4)
	Part-time employee	20 (22.2)
History of smoking	Yes	39 (43.3)
	No	42 (46.7)
	Sometimes	9 (10)
History of psychiatric disorder	Yes	24 (26.7)
	No	66 (73.3)
Psychiatric disorder type	Depression	12 (13.3)
	Anxiety	5 (5.55)
	Both	7 (7.77)
Psychiatric disorder in first degree family members	Yes	12 (13.3)
	No	78 (86.6)
History of suicide	Yes	9 (10)
	No	80 (88.9)
History of suicide in first degree family members	Yes	5 (5.6)
	No	85 (94.4)
History of physical illness	Yes	15 (16.7)
	No	75 (83.3)
History of injecting drug abuse	Yes	1 (1.1)
	No	89 (98.8)
History of substance abuse relapse	Yes	76 (84.4)
	No	14 (15.6)
History of alcohol consumption	Yes	51 (56.7)
	No	39 (43.3)
	No	28 (31.1)
Shireh	Yes	75 (83.3)
	No	15 (16.7)
Opium	Yes	86 (95.6)
	No	4 (4.4)
Cannabis	Yes	16 (17.8)
	No	74 (82.2)
Heroin	Yes	10 (11.1)
	No	80 (88.9)
Amphetamine	Yes	7 (7.8)
	No	83 (92.2)
Methadone/tramadol/ketamine	Yes	10 (11.1)
	No	80 (88.9)

**Figure 1:**
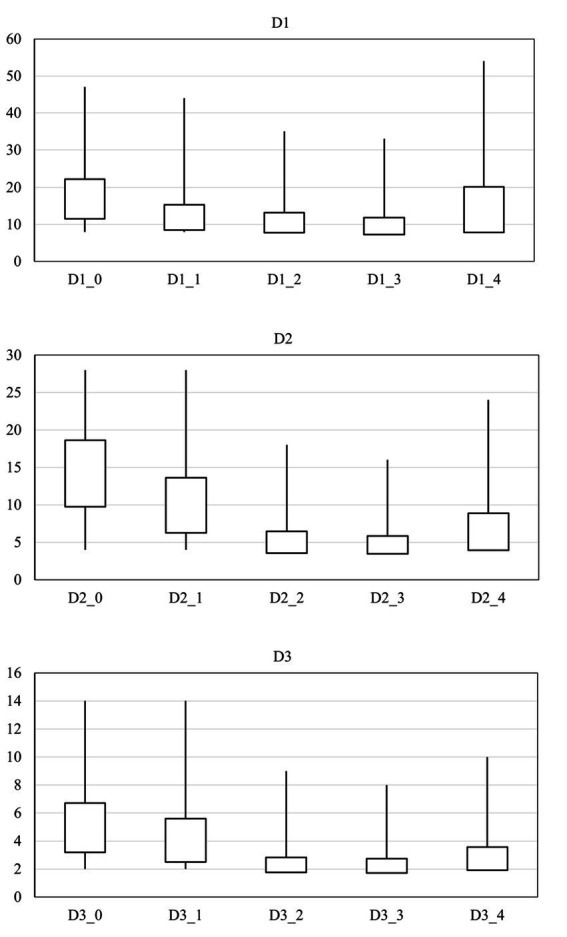
Comparison of the mean scores of craving by time.

Comparison of the mean scores of craving, anxiety, depression, suicide, quality of life, and sleep quality by time are reported in Table 2. Examining the sub-scales of the desire for drug questionnaire (DDQ) showed that the mean range of desire and intention to use drugs, the negative reinforcement range, and the control of drug use were 16.87 ± 10.74, 14.20 ± 8.89, and 4.96 ± 3.51 on arrival. Additionally, it was found that 15 people (16.7%) experienced a relapse during treatment with opium tincture. Therefore, the retention rate was 83.3%. The study also showed a significant difference in desire and intention to use drugs between different times, as the mean score was significantly higher at the beginning of the treatment compared to the end and 1 month after the treatment (*p* = 0.00). Furthermore, the median score of the negative reinforcement was significantly higher at the beginning of the treatment than at the end of the treatment (*p* = 0.00). The score of the substance use control was also significantly lower at the end of treatment (*p* = 0.00), indicating an overall decrease in desire for substance use (Figure 1).

**Table 2:** Comparison of the mean scores of craving, anxiety, depression, suicide, quality of life, and sleep quality by time.

Time	Upon arrival	One month after treatment	5 months after treatment	End of treatment	One month after the end of treatment	*p*-value
DDQ1	12.5 (8–22.3)	9 (8–12.3)	8 (8–11)	8 (8–9)	8 (8–12)	**<0.001**
DDQ2	12 (5–22)	7 (4–16)	4 (4–4)	4 (4–4)	4 (4–6)	**<0.001**
DDQ3	3 (2–8)	2 (2–6)	2 (2–2)	2 (2–2)	2 (2–3)	**<0.001**
Anxiety	8 (2–25.12)	6 (2–25.12)	5.3 (1–25.1)	3 (0–7)	–	**<0.001**
Depression	13.5 (5–26)	6.5 (2.75–16)	3 (1–14.75)	2 (0–8)	–	**<0.001**
Suicide	0 (0–1)	0 (0–0)	0 (0–1)	0 (0–0)	–	0.06
Q1	14 (11.3–16.6)	14.9 (12.6–17.2)	15.2 (12.6–16.6)	16 (13.2–17.2)	–	**0.01**
Q2	12.7 (10–15.4)	14.7 (12–16.7)	15.4 (12.2–17.4)	16.7 (12.7–17.4)	–	**<0.001**
Q3	13.4 (10.7–16)	13.4 (12–16)	14.7 (13.4–16)	16 (12–17.4)	–	**0.01**
Q4	14 (12.9–15.5)	15 (13.5–16)	15 (13.5–16)	15.5 (13–16.5)	–	**0.007**
P1	1 (1–2)	1 (1–2)	1 (1–2)	1 (0–1)	–	0.058
P2	1 (0–2)	1 (1–2)	1 (1–2)	1 (1–2)	–	0.31
P3	1 (0–2)	1 (0–2)	0 (0–1)	0 (0–1)	–	0.07
P4	1 (0–2)	0 (0–2)	0 (0–1)	0 (0–0.5)	–	**0.008**
P5	1 (1–1)	1 (0.8–1)	0.5 (0–1)	0 (0–1)	–	**<0.001**
P6	0 (0–0)	0 (0–0)	0 (0–0)	0 (0–0)	–	**0.02**
P7	1 (0–2)	1 (0–2)	0 (0–1)	0 (0–1)	–	**0.01**
GSI	6 (3–9.3)	6 (4–9)	4 (3–8)	4 (2–6)		**0.001**

Bold numbers are statistically significant.

**Figure 2:**
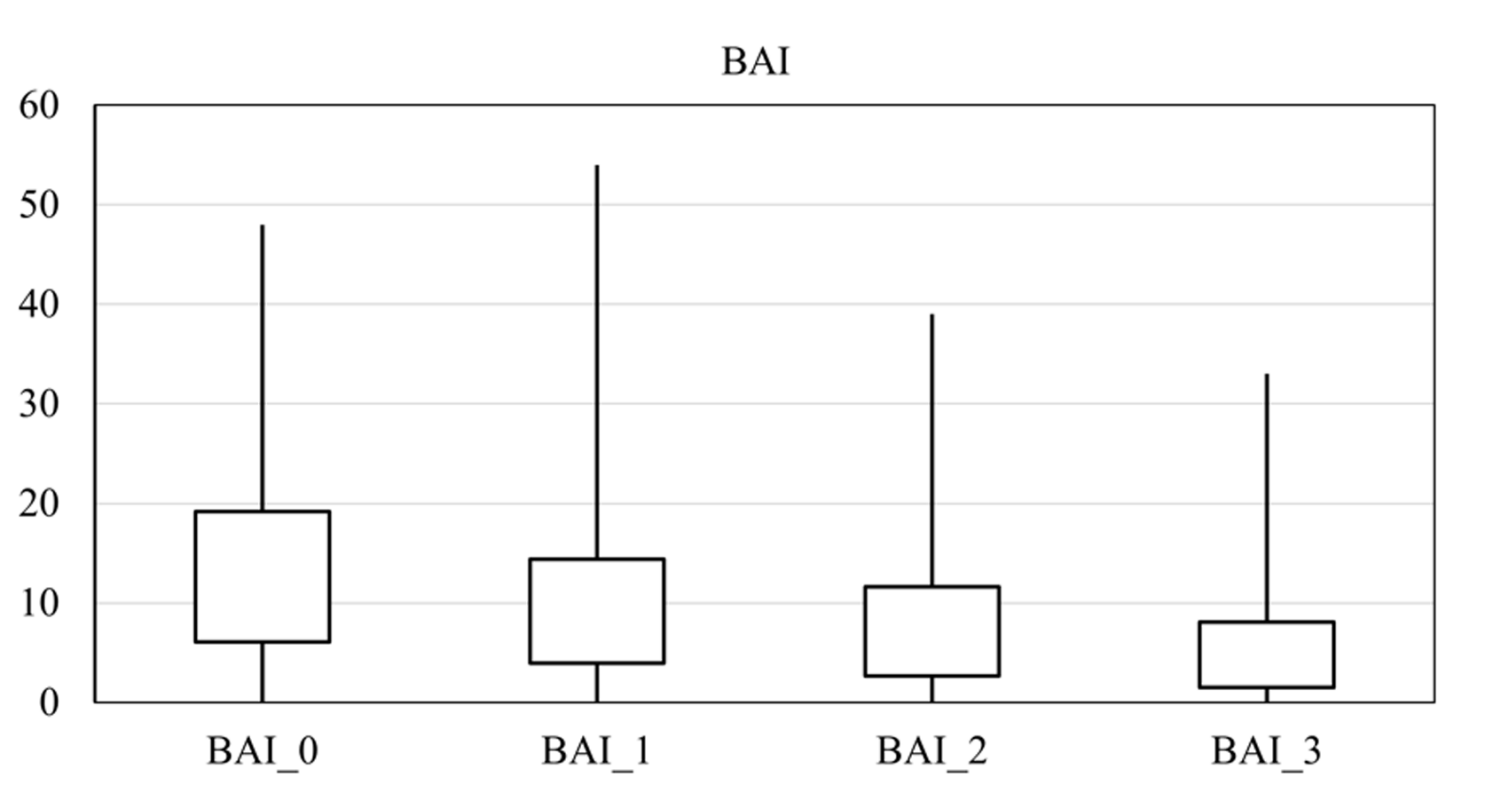
Comparison of the mean scores of anxiety by time.

**Figure 3:**
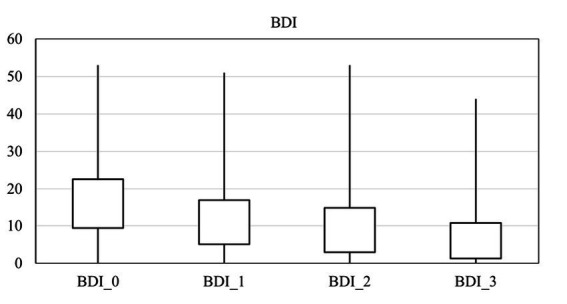
Comparison of the mean scores of depression by time.

The average score for depression, anxiety, and suicide were 16.00 ± 13.19, 13.14 ± 12.62, and 3.39 ± 1.16 on arrival, respectively. There was a significant difference in the anxiety score at different times (*p* = 0.00), and it decreased over time (Figure 2). Also, there was a significant difference in the depression score at different times (*p* = 0.00), and it fell over time (Figure 3). However, it should be pointed out that suicidal thoughts showed no significant difference over time. To interpret this result, one could deduce, that general negative thoughts (anxiety and depression) decreased, but there were no significant changes in the extreme thoughts (suicidal thoughts).

At the time of arrival, the overall score of physical health, mental health, social relationships, and environmental health was 13.62 ± 3.37, 12.40 ± 3.72, 12.93 ± 2.92, and 12.93 ± 2.13. There was a significant difference in physical health between different times, and at the end of the treatment, it was significantly higher than at the beginning (*p* = 0.01). The mental health (*p* = 0.01), social relations (*p* = 0.000), and environmental health (*p* = 0.007) scores were also significantly higher (better) at the end of the treatment.

The overall sleep quality score on arrival was 6.91 ± 4.54, with a significant difference between times (*p* = 0.001), as it decreased significantly over time.

Two by two comparisons between times in all craving scores are shown in Table 3. There was a significant decrease in all areas of craving after 5 months from the start of treatment. In the comparison between the end of the treatment and 1 month after the end of the treatment, there was a significant difference only in negative reinforcement.

**Table 3:** Two by two comparison of craving between times.

Time	Variable	One month after treatment	5 months after treatment	End of treatment	One month after the end of treatment
Upon arrival	DDQ1	0.39	**0.01**	**<0.001**	0.35
	DDQ2	0.99	**<0.001**	**<0.001**	**0.02**
	DDQ3	0.99	**0.003**	**0.001**	0.21
One month after treatment	DDQ1	–	0.99	0.17	0.99
	DDQ2	–	**0.003**	**0.001**	0.99
	DDQ3	–	0.07	**0.01**	0.99
5 months after treatment	DDQ1	–	–	0.99	0.99
	DDQ2	–	–	0.99	0.43
	DDQ3	–	–	0.99	0.99
End of treatment	DDQ1	–	–	–	0.19
	DDQ2	–	–	–	0.14
	DDQ3	–	–	–	0.79

Bold numbers are statistically significant.

Two-by-two comparisons between points in time in all anxiety, depression, and the GSI scores are shown in Table 4. The comparison of the mean between two different times showed that anxiety decreased significantly at the end of treatment and depression decreased immediately (1 month after the start of treatment). Sleep disorders were improved considerably at the end of the treatment.

**Table 4:** Two by two comparison of anxiety, depression, and the GSI between times.

Time	Variable	One month after treatment	5 months after treatment	End of treatment
Upon arrival	Anxiety	0.16	0.068	**<0.001**
	Depression	**0.03**	**0.001**	**<0.001**
	GSI	0.999	0.34	**0.015**
One month after treatment	Anxiety	–	0.99	0.15
	Depression	–	0.99	0.25
	GSI	–	0.25	**0.01**
5 months after treatment	Anxiety	–	–	0.33
	Depression	–	–	0.99
	GSI	–	–	0.99

Bold numbers are statistically significant.

## Discussion

Chronic drug use is associated with strong drug cravings, increasing the vulnerability to addiction. One of the characteristics of opioid use disorder is long-term craving that continues even after detoxification, leading to relapse and reducing maintenance in the treatment process (29, 30). To better manage cravings in treatment retention, previous studies have recommended using effective techniques in substance users (31, 32). In this regard, the present study was designed to investigate the level of craving and retention in the treatment process in patients who undergo structured treatment of gradual detoxification with opium tincture. The findings indicated a decreasing trend in craving scores and an increasing tendency to achieve a better quality of life and sleep over time in patients undergoing opium tincture maintenance therapy. However, it should be noted that 16.7% of the study population indicated relapse during treatment. Therefore, the retention rate with opium-tincture assisted treatment was 83.3%.

The present study found a relapse rate of 16.7% in opium addict patients under opium tincture therapy during the one-year follow-up, which is relatively low compared to other investigations, with relapse rates from 29 to 75% using different protocols and follow-up duration (33–35). Although methadone was identified to be better than opium tincture in keeping patients in treatment, patients’ self-reported drug use was lower with opium tincture (36). A survey on Iranian with substance use disorder has reported that 28.6% of subjects referred to drug rehabilitation centers had a history of drug rehabilitation, and a higher relapse rate was observed in patients who used modern drugs than traditional ones (34). Compulsory detoxification indicated an overall relapse rate of 47.6% in drug abstainers, with a survival time of 220 days to relapse (37). Comparing opium tincture and methadone in a phase III clinical trial has also indicated a lack of non-inferiority of opium tincture to methadone for retaining patients in opioid agonist-used treatment (36). Another cross-sectional study reported the retention rate with methadone and opium tincture, and buprenorphine to be 84.5, 71.8, and 90.1%, respectively, and opium tincture performed significantly better (38). These findings showed various relapse rates and controversial comparisons between studies, likely due to population variations, substance type, treatment procedure, relapse tests, and follow-up time. Additionally, group therapy and motivation programs in the Congress 60 protocol performed in the present study are likely to impact relapse rates. However, a longer duration of follow-up is necessary to confirm this hypothesis.

Craving is a compulsively seeking and abrupt urge to consume the target substance, and it is considered a major risk factor in substance use disorders, as it involves developing and continuing substance abuse, a lack of treatment retention, and repetitive relapses. Therefore, evaluating the effectiveness of addiction treatments often involves assessing their ability to reduce cravings. In the present study, the treatment with opium tincture was found to decrease cravings after 5 months. However, the reduction in craving plateaued 1 month after the end of therapy in DDQ, indicating uncertainty about whether the decline will persist or relapse may occur. As a result, a follow-up study of more than 1 year to ensure retention in treatment without craving and relapse is necessary to confirm the therapy successfully reduces craving and prevents relapse in longer terms. In line with the results of the present study, a systematic review confirmed the effectiveness of opium tincture as adjunctive therapy in treating withdrawal symptoms, maintenance therapy, and efficacy with gradual dose reduction (39, 40). Also, comparing the effectiveness of opium tincture with methadone in treating withdrawal symptoms showed a significant improvement in both methods and no significant difference between them. Considering that the opium tincture is an alcoholic extract of opium and 1 or 2% morphine, therefore it is an effective substance in reducing the symptoms of relapse and craving to prevent the consumption of opium compounds. In general, most studies have proven the effectiveness of methadone in this regard. However, due to the easier access to opium in the Middle East, the acceptability of opium consumption in Iranian culture, and its easy use and low price, opium is a good alternative for drug treatment (40, 41).

Although there is evidence of a correlation between craving and anxiety/depression, it is unclear which causes the other. However, craving and co-occurring psychiatric disorders are associated with a higher risk of relapse. Therefore, understanding the cause-and-effect relationship between craving and mental health can help manage retention in maintenance therapy of substance use disorders. Based on the present study, craving, anxiety, and depression profiles and sleep and life quality were found to be improved following opium tincture therapy. Similarly, a systematic study reported the potential of using opium tincture for retention in treatment, control of withdrawal and craving, enhanced quality of life, and cost-effectiveness for treating opioid use disorder (9). However, these findings are inconclusive due to the lack of active control. Another study investigating the effect of methadone and opium tincture on the sexual performance of opium-dependent patients with a three-month follow-up showed that depression was significantly reduced in the opium tincture group. Anxiety was greatly improved in both treatment groups, and opium tincture showed a more significant reduction than methadone (42). A comparison of psychiatric diseases in opium-dependent patients undergoing maintenance treatment with buprenorphine, methadone, and opium tincture showed that sleep disorders in patients treated with buprenorphine and anxiety in patients treated with methadone were less than in other groups. Also, no difference was found between the three groups of patients in the two subscales of depression and sexual function (43). Investigating the effectiveness of opium tincture on sleep quality in opium-dependent patients also showed that sleep quality, sleep duration, sleep flow, and difficulty in daily functioning in treatment with opium tincture provide better results than buprenorphine and methadone (44). Treatment with alternative methods of opium prevents the recurrence of opium use and improves hormonal pathways, stabilizes hormonal status, and improves symptoms of depression and anxiety in these patients. Considering the preference of opioid complete agonists to the μ receptor, the effectiveness of opium tincture on sleep can be justified. These results highlight that treatment with opium tincture can significantly reduce comorbidities despite the continued use of opium. A systematic review also reported that opioid agonist treatments improve mental health in individuals with opioid use disorder (45).

Maintenance treatment with therapeutic agonists, especially methadone and buprenorphine, is among the most common treatments for patients with high-risk behaviors (46). In expert policies, the purpose of maintenance treatment includes disconnection from the trafficking system and direct connection of patients with health systems, reducing the costs of drug use in patients, reducing the economic pressure on the families, and finally, reducing the crime related to drugs. Therefore, treatment with opium tincture, despite continued opium consumption, can significantly reduce the social damage caused by addiction. Since the duration of detoxification treatments ranges from a few weeks to 6 months, and the time of treatment with opium tincture is 11 months, the treatment with opium tincture is not included in the detoxification category. However, considering that the dosage of opium tincture is reduced during the treatment, this treatment can be viewed as a gradual detoxification. Because in the maintenance treatment, the amount of the consumed substance does not change, and more effective and less potent drugs are also used.

During withdrawal, treatment happens if the patient’s thinking changes; training and group therapy are needed to create this change. As a strong point, in this structured therapy study with the 60th Congress, it has been investigated that modifying the person’s thinking is one of the parts of the treatment and that the mind is balanced along with the body. Also, a one-year follow-up and examination of changes in several psychiatric disorders, including depression, anxiety, suicide, and sleep disorder, has led to a more detailed treatment examination.

However, this study had limitations that should be acknowledged. In addition to the small sample size, the participants were limited to those referred to educational hospitals, which may limit the generalizability of the results to the general population. The study population did not include individuals with severe psychiatric and physical disorders that could affect the results. Additionally, the relapse criteria relied on interviews with patients and their accompanying attendants rather than regular urine screening tests. The participants were followed up for 1 year based on the Congress 60 protocol for opium tincture decreasing to zero; so, craving and relapse were not considered for a longer time after treatment. The COVID-19 pandemic was another study limitation that made it difficult to follow up with the patients and caused the samples to drop. In addition, different types of common opium compounds in Iran, such as Shireh, differ in purity and, as a result, the occurrence of psychiatric symptoms and relapse. Further research using a larger sample size and longer follow-up time is necessary to confirm the reliability of the present study’s findings and the efficacy of opium tincture as a treatment program for opium users. Controlled clinical trials should also be conducted to evaluate the superiority of maintenance therapy procedures against different substances.

## Conclusion

Opioid use disorder is the most common substance use disorder in Iran. Therefore, designing effective interventions for treating patients requires identifying relapse-related factors and their management. This study was the first to examine the rate of relapse and craving for drug use and changes in psychiatric disorders in people with opioid use disorder treated with opium tincture in the form of the 60th Congress. The results indicated decreased depression, anxiety, craving, and increased quality of life and sleep profiles following gradual detoxification with opium tincture under the Congress 60 protocol. Moreover, the relapse rate in a one-year follow-up duration was lower than in previous investigations. These findings highlight the effectiveness of opium tincture treatment for patients with opioid use disorder in controlling craving and improving mental health and life quality, and consequently, lowering relapse rate.

## Data availability statement

The raw data supporting the conclusions of this article will be made available by the authors, without undue reservation.

## Ethics statement

The studies involving humans were approved by the Ethics Committee of Mashhad University of Medical Sciences with the ethical code IR.MUMS.MEDICAL.REC.1399.686, all the study steps were carried out according to ethical protocols. The studies were conducted in accordance with the local legislation and institutional requirements. The participants provided their written informed consent to participate in this study.

## Author contributions

SO: Conceptualization, Investigation, Writing – original draft. AR: Conceptualization, Supervision, Validation, Writing – review & editing. SA: Writing – review & editing, Data curation, Formal analysis, Methodology. MS: Investigation, Writing – original draft. AT: Conceptualization, Project administration, Supervision, Validation, Visualization, Writing – review & editing.
